# Dynamic regulation of *Rgs16* and its correlation with Neuregulin1 expression in acute and chronic nerve injury

**DOI:** 10.3389/fcell.2025.1540453

**Published:** 2025-03-27

**Authors:** Marina García-Bejarano, Riccardo Aucello, Federica Zen, Marwa El Soury, Francesca Cordero, Jesús M. de la Fuente, Isabelle Perroteau, Giulia Ronchi, Giovanna Gambarotta

**Affiliations:** ^1^ Department of Clinical and Biological Sciences (DSCB), University of Torino, Orbassano, Italy; ^2^ Neuroscience Institute Cavalieri Ottolenghi (NICO ), University of Torino, Orbassano, Italy; ^3^ Institute of Nanoscience and Materials of Aragon (INMA), CSIC-University of Zaragoza and CIBER-BBN, Zaragoza, Spain; ^4^ Computer Science Department, University of Torino, Torino, Italy

**Keywords:** peripheral nerve injury, single-cell RNA sequencing, regulator of G protein signaling 16, Neuregulin1, chronic demyelinating neuropathy, neuropathic pain, CMT1A

## Abstract

**Introduction:** Regulators of G Protein Signaling (RGS) form a gene family that modulates G protein-coupled receptor signaling by enhancing the GTPase activity of the Gα-GTP complex, effectively inhibiting G protein-dependent signal transduction cascades. While RGSs are expressed across many organs, including the central nervous system, few data are available for the peripheral nervous system (PNS).

**Methods and Results:** To investigate potential links between RGS and PNS, open-access single-cell RNA sequencing datasets were analyzed, focusing on mice intact sciatic nerves and distal stumps at 3 and 9 days post-transection. *Rgs16* emerged as the RGS member most highly expressed by Schwann cells after injury, suggesting its involvement in nerve degeneration. To further explore *Rgs16* behavior in nerve injury, its expression was assessed at mRNA level at different time points in the median nerve of adult rats under regenerating conditions following mild (crush) or more severe (end-to-end repair) traumatic injury, and under degenerating conditions. Results revealed that *Rgs16* expression increased 3 days after injury, declining under regenerating conditions, but remaining high in degenerating conditions. To examine the role of *Rgs16* in chronic nerve degeneration, its expression was evaluated in a pathological model of Charcot-Marie-Tooth disease type-1A (CMT1A), a chronic demyelinating peripheral neuropathy. Analysis of publicly available RNA sequencing data from sciatic nerves of wild-type and CMT1A rats during development showed a significant upregulation of *Rgs16* in transgenic rats at P18. Interestingly, this upregulation mirrored the expression pattern of Neuregulin1 (*Nrg1*), a gene critical for Schwann cell dedifferentiation and demyelination, strongly upregulated in traumatic and chronic nerve injuries. To explore a potential NRG1-RGS16 link, primary Schwann cell cultures were treated with recombinant NRG1β1, which induced an increase in *Rgs16* expression.

**Discussion:** These findings suggest a potential feedback mechanism where transient *Rgs16* upregulation in response to injury and/or NRG1 might negatively regulate NRG1 release through RGS16-mediated inhibition of GPCR/ErbB transactivation. This study highlights the dynamic role of *Rgs16* in traumatic and chronic nerve injuries, implicating its involvement in processes of nerve degeneration, regeneration, and possibly neuropathic pain. Further investigations are needed to clarify RGS16 function, which could pave the way for novel therapeutic strategies to enhance nerve regeneration and alleviate neuropathic pain.

## 1 Introduction

G protein-coupled receptors (GPCRs) are among the most crucial transmembrane proteins in nature, orchestrating a vast array of cellular signaling pathways and regulating numerous biological processes (Lambert, 2008; Tian et al., 2022). These processes include the detection of light, odors, hormones, and neurotransmitters (Lambert, 2008; Soundararajan et al., 2008). In their inactive state, G proteins exist as a heterotrimeric complex consisting of the Gα subunit bound to guanosine diphosphate (GDP) and tightly associated with the Gβγ dimer. This complex is coupled to the GPCR. Upon activation by chemical or physical stimuli, GPCRs undergo conformational changes, which reduce the affinity of the Gα subunit for GDP, leading to its release. The high intracellular concentration of guanosine triphosphate (GTP) ensures rapid binding to the now vacated nucleotide-binding site, transitioning the Gα subunit into its active form (Neer, 1995). GTP binding induces structural changes in the Gα subunit, causing dissociation of the Gα-GTP complex from the Gβγ dimer. Both components are now free to regulate downstream effectors, including enzymes and ion channels, thereby initiating a cascade of cellular responses (Wieland and Chen, 1999; Lambert, 2008; Soundararajan et al., 2008; Tian et al., 2022). Depending on the type of Gα subunit—such as Gαs, Gαi/o, Gαq/11, or Gα12/13—different signaling pathways are activated or inhibited, each with distinct physiological outcomes (Hepler, 1999; Larminie et al., 2004; Soundararajan et al., 2008; Tian et al., 2022). This activation persists until the intrinsic GTPase activity of the Gα subunit hydrolyzes GTP to GDP, enabling the reassociation of the Gα, Gβ, and Gγ subunits into the inactive heterotrimer. This reassociation restores the basal state of the signaling system, allowing the cycle to repeat in response to new stimuli (Gilman, 1995; Neer, 1995; Soundararajan et al., 2008). Through their ability to integrate extracellular signals and regulate intracellular responses, GPCRs play a central role in maintaining cellular homeostasis and coordinating complex biological processes.

The intrinsic GTPase activity of the Gα subunit is modulated by proteins known as Regulators of G Protein Signaling (RGS), which accelerate GTP hydrolysis (Wieland and Chen, 1999; Derrien and Druey, 2001; Larminie et al., 2004; Tian et al., 2022; Yuan and Yang, 2022). RGS proteins interact with and activate specific Gα subunits, including members of the Gαi family, Gαq, and Gα12/13, but notably not Gαs (Hepler, 1999; Berthebaud et al., 2005). The human genome encodes 37 proteins possessing at least one conserved canonical RGS domain (Hepler, 1999; Soundararajan et al., 2008). Based on structural characteristics, proteins containing an RGS domain are classified into nine subfamilies: A/RZ (RGS17, -19, −20), B/R4 (RGS1-5, −8, −13, −16, −18, −21), C/R7 (RGS6, -7, -9, -11), D/R12 (RGS10, -12, −14), E/RA (AXIN, AXIN2), F/GEF (p115-RhoGEF, PDZ-RhoGEF, LARG), G/GRK (GRK1-7), H/SNX (SNX13, -14, −25) and I/D-AKAP (RGS22, D-AKAP2) (Bansal et al., 2007; Tian et al., 2022; Yuan and Yang, 2022). Nevertheless, only 20 of them, mainly belonging to the first four subfamilies, modulate Gα GTPase activity (Stewart and Fisher, 2015). RGS proteins are critical regulators of homeostatic processes, ensuring proper cellular signaling and physiological balance. Disruption in their expression or function has been implicated in the pathogenesis of various diseases, including cancer, cardiovascular disorders, and neurodegenerative conditions (Lee and Bou Dagher, 2016; Alqinyah and Hooks, 2018; Yang et al., 2023).

Despite significant progress in understanding their roles across multiple biological systems, the functions of GPCR and RGS proteins in the peripheral nervous system (PNS) remain insufficiently explored. This gap persists even though different GPCRs have been described in Schwann cells, the key players of peripheral nerve regeneration. For instance, Gpr126/Adhesion G protein-coupled receptor G6 (Adgrg6) and Gpr44/Prostaglandin D2 receptor 2 play key roles in Schwann cell development and myelination (Monk et al., 2009; Trimarco et al., 2014). Lysophosphatidic acid receptor 1 (LPA1) is required for Schwann cell survival, radial sorting, proper myelination and neuropathic pain (Weiner and Chun, 1999; Anliker et al., 2013). The γ-aminobutyric acid B (GABA-B) receptors have a role in neuropathic pain alleviation (Magnaghi et al., 2004; Magnaghi et al., 2014). Additionally, CXC chemokine receptor 4 (CXCR4), the receptor of stromal cell-derived factor (SDF1α or CXCL12), is involved in repair Schwann cell migration (Gao et al., 2018).

Furthermore, knowledge about the potential role of RGS proteins in peripheral nerve development and post-injury regeneration remains largely limited. RGS4 is the most studied, together with RGS3 and RGS12. RGS4 is broadly expressed in a dynamic manner during embryonic development in a small set of peripheral neuronal precursors (Grillet et al., 2003) and is upregulated in the lumbar dorsal root ganglia (DRG) and dorsal horn of rats developing tactile hypersensitivity after sciatic nerve lesion (Taccola et al., 2016). RGS4, with RGS3, is constitutively expressed at high levels in C-fiber primary sensory neurons in the adult rat DRG (Costigan et al., 2003). RGS12 downregulation negatively affects nerve growth factor-mediated axonal growth in primary mouse DRG neurons (Willard et al., 2007).

Therefore, this research seeks to investigate whether RGS proteins, which play a key role in GPCR activity, are regulated in the nerve in response to injury. The goal is to deepen our understanding of the molecular mechanisms underlying nerve repair processes, which could potentially open new avenues for therapeutic interventions aimed at enhancing nerve regeneration and functional recovery.

## 2 Materials and methods

### 2.1 Computational analysis of single-cell RNA-seq datasets of mouse injured nerves

#### 2.1.1 Datasets and study design

A comprehensive single-cell RNA sequencing (scRNA-seq) data analysis was conducted to investigate cellular heterogeneity in intact and injured peripheral nerves from adult mice of both sexes. The study aimed to evaluate the expression patterns of the *Rgs* gene family across various cell types (annotated clusters) and conditions by integrating datasets to analyze cells of the same type under different scenarios. Special emphasis was placed on Schwann cells, with a detailed investigation into the expression dynamics of *Rgs16*, providing insights into its potential role in nerve injury and repair.

GSM4423509 (adult mouse uninjured sciatic nerve, [Un] (Toma et al., 2020)), GSM4423506 (distal portion of adult mouse sciatic nerves, 3 days post-transection [3d] (Toma et al., 2020)), and GSM3408138 (distal portion of adult mouse sciatic nerves, 9 days post-transection [9d] (Carr et al., 2019)) were downloaded from the NCBI GEO database. The analysis of these datasets was conducted using Seurat v.5.1.0 (https://satijalab.org/seurat/).

Clustering and annotation procedures were executed, allowing for the accurate mapping of critical cell types. The downstream analyses included the identification of cell type-specific gene expression markers across clusters. Following scRNA-seq data quality control of the three datasets, the focus was directed toward the expression profiles of the *Rgs* gene family across clusters for each dataset, with a particular emphasis on Schwann cells.

Subsequently, data from different conditions were integrated to perform binary comparative analyses between Un and 3d, and between Un and 9d. These datasets, containing treatment information (Un vs. 3d and Un vs. 9d), allowed for investigating gene expression changes in different conditions for cells of the same type. Differentially expressed genes (DEGs) were identified by comparing each condition. DEG analysis was conducted within the same cell type (Schwann cells) across conditions (inter-dataset), focusing on the *Rgs* gene family, including *Rgs16*. The statistical significance was assessed by computing adjusted p-values using the Wilcoxon Rank Sum test and the Likelihood-ratio test.

#### 2.1.2 Single-cell RNA sequencing data processing

Raw sequencing data were processed in the R environment (R 4.4.0) by the following steps.

##### 2.1.2.1 Quality control

Quality control was performed to filter out low-quality cells and retain high-quality data for downstream analyses (Supplementary Figure S1). Standard quality control metrics were used, including the number of Unique Molecular Identifiers (UMI), the number of genes per cell, the mitochondrial content, and the ratio of genes per UMI. Cells were filtered based on UMI counts (500–7,500), number of genes detected (750–5,000), gene complexity (log10(GenesPerUMI) > 0.8), and mitochondrial reads (<10%). These steps ensured robust downstream analysis of high-quality single-cell transcriptomes, suitable for clustering, differential expression analysis, and gene expression characterization, including the *Rgs* gene family.

##### 2.1.2.2 Normalization

Gene expression counts from each scRNA-seq dataset were normalized to account for differences in sequencing depth and prepare the data for downstream analysis. Seurat’s *LogNormalize* method was employed to normalize the gene expression counts for each cell by scaling the total UMI count per cell, followed by a log transformation. A scaling factor (sf = 10,000) was used to adjust for differences in sequencing depth across cells.

Seurat’s *FindVariableFeatures* identified highly variable genes across cells using the variance-stabilizing transformation (VST) method. This step allowed the identification of the top variable genes, which are likely biologically informative. The normalized expression values were scaled and centered using the *ScaleData* function. This step helps shift the expression of each gene so that the mean expression across cells is zero and reduces the impact of outliers, ensuring a consistent range of expression values across cells.

To minimize technical artifacts, Seurat’s *ScaleData* was exploited to regress the number of UMIs and the proportion of mitochondrial genes, ensuring that unwanted sources of variation (i.e., differences in sequencing depth or mitochondrial content) do not confound the downstream analysis. Finally, a Principal Component Analysis (PCA) was conducted to reduce the dimensionality of the dataset, selecting Principal Components (PCs) that accounted for more than 90% of the variance but contributed less than 5% each for further analysis. The normalized and scaled data were then utilized for clustering and differential gene expression analysis.

#### 2.1.3 Clustering

Clustering was performed on the 3 mouse peripheral nerve datasets to identify distinct cell populations. The selected significant PCs were used to construct a k-nearest neighbor (kNN) graph based on the PCA space. This step exploits Seurat’s *FindNeighbors* to identify the neighboring cells that are most similar based on their PCA scores. Cell clustering was then performed using the Louvain algorithm through the *FindClusters* function, with the resolution parameter set to 1.5, as recommended for datasets comprising 1,000 to 5,000 cells. The resolution parameter controls the granularity of the clusters, with higher values resulting in a higher number of smaller clusters. The resolution parameter was set to capture biologically relevant cell populations for clustering purposes while maintaining a balance in cluster granularity.

To visualize the clusters in a lower-dimensional space, t-distributed Stochastic Neighbor Embedding (t-SNE) was employed, with the perplexity parameter set to 40. This value helps to balance local *versus* global relationships in the data and is typically used for datasets containing 1,000–5,000 cells. These clusters were subsequently utilized for further differential expression and cell-type-specific analyses across datasets.

#### 2.1.4 Cell type annotation

A curated list of marker genes from the *PanglaoDB* database (Franzén et al., 2019) (last updated 27 March 2020) was utilized to guide cell type identification. The database contains well-established marker genes for various cell types across mice and human species. Only marker genes annotated for mice or shared between mice and humans were retained. Following the clustering of cells using the Seurat package, the DEGs for each cluster were matched to the marker genes from *PanglaoDB* to assign cell types. DEGs were identified using Seurat’s *FindAllMarkers* function (rely on the “Intra-dataset Gene Expression Analysis” subsection for further detail).

DEGs for each cluster were compared to the marker genes in *PanglaoDB*. The top 3 cell types with the highest number of shared marker genes were identified for each cluster. These cell types were used to annotate each cluster, with the cell type having the highest number of matching markers assigned as the primary label. Schwann cells were specifically identified based on the presence of known Schwann cell marker genes. Clusters exhibiting significant overlap with Schwann cell markers were annotated accordingly. The annotated clusters and their respective cell types were saved for subsequent analysis and visualization.

#### 2.1.5 Gene expression analysis

Intra-dataset gene expression analysis was conducted to characterize gene expression profiles and identify cell type-specific markers within each dataset. Additionally, inter-dataset gene expression analysis was performed to characterize the gene expression profiles of *Rgs* family members in Schwann cells across the datasets.

##### 2.1.5.1 Intra-dataset gene expression analysis

Gene expression profiles across clusters were characterized within each dataset. The expression of the *Rgs* gene family was specifically assessed across clusters, focusing on Schwann cells.

At first Seurat’s *FindMarkers* function was employed for each identified cluster to identify DEGs within the cluster compared to the rest of the dataset. A gene was considered a marker if at least 10% of the cells in the cluster expressed it. Only genes with an adjusted p-value (Bonferroni-corrected) less than 0.05 were considered statistically significant. Moreover, a log-fold change threshold of 0.25 was applied. The top 12 genes with the highest absolute values of log2 fold change were selected as markers for each cluster.

To identify global markers across all clusters within each dataset, Seurat’s *FindAllMarkers* function was used. This approach allowed for the identification of the most distinctively expressed genes across all clusters. The following criteria were applied: at least 10% of cells in a cluster must express the gene, and a log-fold change threshold of 0.25 was needed to filter out genes with minimal expression differences.

##### 2.1.5.2 Inter-dataset comparative analysis

To investigate the expression dynamics of the *Rgs* gene family across different conditions in mouse peripheral nerve cells, an inter-dataset comparative analysis was performed on scRNA-seq data derived from intact and injured nerve tissues. Binary comparative analyses were performed between conditions: (i) Un x 3d and (ii) Un x 9d. Cell type comparisons were restricted to Schwann cells, ensuring that clusters corresponded to the same cell type across datasets.

Canonical correlation analysis (CCA) was employed via Seurat’s *FindIntegrationAnchors* function to integrate the datasets. Subsequently, Seurat’s *IntegrateData* function was applied to combine the datasets into a single integrated Seurat object for downstream comparative analysis. Clustering was conducted using Seurat’s *FindNeighbors* function on the first 10 PCs, followed by *FindClusters* with a resolution parameter of 0.80 to identify distinct cell populations. Finally, t-SNE was applied for cluster visualization, using a perplexity setting of 50 to balance local and global cluster representation.

#### 2.1.6 Differential expression analysis

Differential expression analysis was performed using the Seurat R package to identify DEGs across experimental conditions. To investigate the impact of peripheral nerve injury on gene expression, comparisons were made between intact (Un) and injured mouse sciatic nerves at two post-injury time points: 3d and 9d. The Un served as the control group. Each dataset was processed independently, followed by integration to facilitate cross-condition comparisons.

##### 2.1.6.1 Identification of differentially expressed genes (DEGs)

Seurat’s *FindMarkers* function was applied to identify DEGs between experimental conditions. The differential expression analysis was assessed by comparing Schwann cells, setting the intact condition as first identity and the injured condition as second identity.

The Wilcoxon rank-sum test was used as the default statistical test, with an adjusted p-value threshold of 0.05 to account for multiple comparisons via Bonferroni correction. Further investigation was conducted on genes showing significant differences in the default Wilcoxon rank-sum test and the bimodal likelihood-ratio test. Both tests demonstrate thoroughness and an attempt to ensure robustness in the findings. To focus on the *Rgs* gene family, the results were filtered to focus on the expression of these genes across conditions. The expression status of *Rgs* family genes, including *Rgs16*, was assessed in Schwann cells. The distribution of gene expression warranted using the Likelihood-ratio test for single-cell gene expression (McDavid et al., 2013) and provides a more sensitive analysis in this context.

Genes were considered DE if they exhibited a logFC greater than 0.1 and had a minimum expression in at least 10% of the cells. DEGs were filtered based on an adjusted p-value threshold of 0.05.

#### 2.1.7 Datasets and code availability

The scRNA-seq datasets used in this study are publicly available and can be accessed at the following links: (i) GSM4423509, (ii) GSM4423506, and (iii) GSM3408138. The datasets were last downloaded on 20 Jan 2024. Any additional information required to reanalyze the data reported here is available upon request.

### 2.2 Computational analysis of single-cell RNA-seq dataset of rat injured nerves

#### 2.2.1 Dataset and study design

scRNA-seq data analysis was conducted to investigate *Rgs* expression in rat Schwann cells after injury. The dataset GSE216665 (Lovatt et al., 2022), downloaded from the NCBI GEO database, comprises over 97,000 cells derived from 25 adult rat sciatic nerve tissue samples: uninjured sciatic nerve [Un]: 12,636 cells; 3 days post-chronic constriction injury [3d]: 22,288 cells; 12 days post-chronic constriction injury [12d]: 62,144 cells.

#### 2.2.2 Single-cell RNA sequencing data processing

Raw sequencing data were processed in the R environment (R 4.4.0) by the following steps.

##### 2.2.2.1 Quality control

Cells with fewer than 200 detected genes are excluded to filter out empty droplets and low-quality cells. The upper threshold of 5,500 genes prevents the inclusion of potential doublets, ensuring that only single cells are retained. The percentage of mitochondrial gene expression is calculated per cell as a quality control metric. A threshold of 10% is applied to exclude damaged or dying cells while still allowing for the retention of cells with moderately elevated mitochondrial expression, which may be biologically relevant in stressed conditions such as nerve injury.

##### 2.2.2.2 Normalization

*SCTransform* (SCT) is used for normalization instead of the traditional *LogNormalize* method. Unlike log-based approaches, SCT models the mean-variance relationship of UMI counts and performs variance stabilization. This is advantageous for heterogeneous tissues. Mitochondrial gene expression percentage is included as a variable to regress out during normalization (vars.to.regress = “percent.mt”). This step mitigates technical biases caused by cell stress or damage, ensuring that downstream analyses are not dominated by artifacts of mitochondrial expression variability.

##### 2.2.2.3 Data integration and batch correction

Tissue samples were utilized as the batch variable in *Harmony* since each sample was processed separately. This approach effectively removes technical differences between samples while preserving biological variation between conditions. *Harmony* is designed to correct batch effects in scRNA-seq data while maintaining biological heterogeneity.

#### 2.2.3 Clustering

The selection of the first 30 PCs aligns with the methodology outlined in the original publication (Lovatt et al., 2022) ensuring that the majority of the variance in the data is captured. The *FindNeighbors* function computes the kNN for each cell to identify the neighboring cells based on their similarity in the harmony-corrected space.

#### 2.2.4 Cell type annotation

The annotation of cell types in the scRNA-seq dataset follows a marker-based approach leveraging established gene expression signatures. A comprehensive scoring system is employed to match the clusters with cell types.

Calculation of marker expression methodology employs a scoring system to match clusters with cell types. For each marker gene in each cell type the percentage of cells expressing the marker in each cluster was calculated and the mean expression level across all cells in the cluster was determined.

To compute the scores for each cell type-cluster combination (i) the percentage of marker genes expressed above a threshold (10%) was calculated, (ii) the average expression level across all markers was calculated and the average percentage of cells expressing any marker was determined.

This multi-faceted scoring approach ensures that assignments are based on both marker breadth (how many markers are expressed) and depth (expression levels and prevalence) via assignment of confidence levels: *High*, *Medium*, *Low*, and *Very low*.

Schwann cells were isolated by subsetting cells classified as “Schwann cells” based on established marker genes. A confidence level of *High* was assigned to the cluster annotated as ‘Schwann cells’ which is composed of 16,969 cells.

#### 2.2.5 *Rgs* gene family analysis

For targeted analysis of the *Rgs* gene family, *Rgs* gene expression patterns and regulation in Schwann cells following chronic constriction injury was explored. To obtain expression profiling across conditions, average expression levels of all *Rgs* family genes were calculated across experimental conditions (Un, 3d, 12d) using SCT-normalized data. This allowed visualization of expression trends for all *Rgs* genes, facilitating the identification of condition-specific patterns. Expression profiling and differential expression analysis of *Rgs* genes within the Schwann cell population were performed using Wilcoxon and bimodal tests (adjusted p-value [B-H procedure] ≤ 0.001 |log_2_FC| ≥ 0.5). Filtering criteria were applied (minimum percentage in either population ≥ 12.5%.

### 2.3 Computational analysis of RNA-seq dataset of Charcot-Marie-Tooth-1A affected rat nerves

Processed RNA-sequencing (RNA-seq) data of sciatic nerves derived from wild-type and Pmp22 transgenic rats (Charcot-Marie-Tooth disease type-1A models) at embryonic day 21 (E21), postnatal day 6 (P6) and 18 (P18) were obtained from the GSE115930 dataset available in the GEO database (Fledrich et al., 2018) (n = 4 for each condition). Normalization of RNA-seq read counts was performed with DESeq2 v1.40.2 (Love et al., 2014) to account for non-biological variations between samples, such as those arising from library preparation, sequencing depth, gene length, mapping biases, and other technical factors (Li et al., 2020). The average count was calculated, displaying the total reads obtained for each condition and gene.

### 2.4 Peripheral nerve surgery

In this study, no new animals were subjected to experimental procedures; instead, biological samples obtained from a previous study were used (Ronchi et al., 2016), in line with the ethical principles of the 3Rs (Replacement, Reduction, and Refinement), allowing us to maximize the use of available biological material, minimize the need for additional animal subjects, and uphold rigorous ethical standards in scientific research. The samples used in this work derived from adult female Wistar rats that had undergone specific nerve injury and repair procedures as part of prior experiments, already described in Ronchi et al., 2016, including: (i) Control group: uninjured median nerves. (ii) Crush injury (axonotmesis) group: median nerves were crushed at the mid-level of the humerus using a non-serrated clamp exerting a compression force of 17.02 MPa for a duration of 30 s, representing a moderate peripheral nerve injury model, where the axons are damaged, but the connective tissue framework remains intact (Ronchi et al., 2009). (iii) End-to-end repair (neurotmesis) group: median nerves were transected at the mid-level of the humerus, followed by immediate reconnection of proximal and distal nerve stumps using two epineural sutures (9/0), representing a severe nerve injury model, where both axons and connective tissue are disrupted. (iv) Degenerated nerve group: median nerves were transected at the mid-level of the humerus, with the distal nerve stump left unrepaired, and the proximal stump sutured to the pectoralis major muscle to prevent regeneration, representing a chronic degeneration model. For mRNA and protein analysis, the healthy median nerves of the control group and the distal portion of the injured median nerves were collected from three animals (n = 3) per time point post-injury (3, 7, 14, and 28 days).

### 2.5 Primary Schwann cell culture and stimulation with recombinant NRG1β1

To obtain primary Schwann cell cultures, sciatic nerves were collected from adult female Wistar rats. The epineurium was carefully removed, the nerves were sectioned into smaller fragments and cultured in Dulbecco Modified Eagle Medium (DMEM, Gibco, Thermo Fisher Scientific, Waltham, MA, United States) containing 1 g/L glucose, 10% heat-inactivated fetal bovine serum (FBS; Invitrogen, Thermo Fisher Scientific), 100 units/mL penicillin, 100 μg/mL streptomycin. Each cell culture was obtained from two sciatic nerves of the same rat. After 1 week of incubation at 37 °C in a 5% CO_2_ atmosphere saturated with H_2_O, the fragments were transferred to a 3 cm Petri dish and incubated with dissociation medium containing Collagenase IV and Dispase II enzymes. Following 24 h of enzymatic digestion, the tissue underwent mechanical dissociation; the medium containing the dissociated nerves was collected in a tube, then the suspension was filtered through a cell strainer with 70 µm pores (Sartorius Stedim Biotech GmbH, Göttingen, Germany) and transferred into a new tube. Cells were centrifuged at 100 rcf for 5 min and then resuspended in a selective DMEM D-valine medium (AL251-500ML; HiMedia Laboratories, Thane, India) enriched with 10% FBS, 100 μg/mL streptomycin, 100 U/mL penicillin, 10 µM forskolin and 8 nM NRG1β1 (#396-HB, R&D Systems, Minneapolis, United States) and plated onto poly-L-lysine (PLL) coated culture dishes. The obtained primary Schwann cell culture was maintained at 37 °C in a 5% CO_2_ atmosphere saturated with H_2_O. The cells (passage 1) were allowed to proliferate until confluency and then transferred to a 6 cm culture dish, allowing them to reach confluency (passage 2). Subsequently, cells were passed to a 10 cm culture dish (passage 3); for the following passages cells were diluted 1:2. The experiments were performed from passages 4 to 10. To assess whether NRG1β1 regulates RGS16 expression, cells were starved for 18 h in a medium containing 2% FBS - in the absence of NRG1β1 - to minimize basal signaling. The following day, cells were treated with 10 nM NRG1β1 for 15, 30 and 60 min, 3, 6 and 24 h. At each time point, RNA and proteins were extracted.

### 2.6 RNA isolation, cDNA preparation and quantitative Real-Time Polymerase Chain Reaction

Total RNA was extracted using TRIzol reagent (Invitrogen, Massachusetts, United States) according to the manufacturer’s instructions. Reverse transcription was performed employing 250 ng of total RNA in a 25 µL reaction volume containing: 1x RT-Buffer (ThermoFisher Scientific, Vilnius, Lithuania), 0.1 μg/μL bovine serum albumin (BSA; Sigma Aldrich, Missouri, United States), 0.05% Triton X-100 (Sigma Aldrich), 1 mM dNTP (R0192; ThermoFisher Scientific), 7.5 µM random hexamer primers (SO142; ThermoFisher Scientific), 40 U RiboLock RNAse Inhibitor (EO0381; ThermoFisher Scientific) and 200 U RevertAid Reverse Transcriptase (EP0441; ThermoFisher Scientific). The reaction was carried out at 25 °C for 10 min, 42 °C for 90 min, 70 °C for 10 min and 12 °C for 20 min, using the GeneAmp® PCR System 9,700 (Applied Biosystems, Life Technologies Europe BV, Monza, Italy). The cDNA diluted 10-fold in nuclease-free water was analyzed by Real-Time Polymerase Chain Reaction (qRT-PCR) in a 20 µL reaction volume with 1x iTaq Universal SYBR Green Supermix (BioRad, California, United States) and 300 nM each of forward and reverse primers. Primers for Ankyrin Repeat Domain-containing Protein 27 (*Ankrd27*), RPTOR Independent Companion of MTOR Complex 2 (*Rictor*) and TATA box binding protein (*Tbp*) were previously designed (Gambarotta et al., 2014). Primers for *Rgs16* were designed using AnnHyb software (http://www.bioinformatics.org/annhyb/) and synthesized by Invitrogen (Life Technologies Europe BV, Monza, Italy). The information regarding all the primers is described below: *Ankrd27* (accession number #NM_001271264; amplicon length 95 pb; *Ankrd27* forward: 5′-CCAGGATCCGAGAGGTGCTGTC-3′, *Ankrd27* reverse: 5′- CAGAGCCATATGGACTTCAGGGGG-3′); *Rictor* (accession number #XM_001055633; amplicon length 81 pb; *Rictor* forward: 5′-GAGGTGGAGAGGACACAAGCCC-3′, *Rictor* reverse: 5′-GGCCACAGAACTCGGAAACAAGG-3′); *Tbp* (accession number #NM_013684.3; amplicon length 106 pb; *Tbp* forward: 5′-GATCAAACCCAGAATTGTTCTCC-3′, *Tbp* reverse: 5′-GGGGTAGATGTTTTCAAATGCTTC-3′) and *Rgs16* (accession number #NM_011267.3; amplicon length 133 pb; *Rgs16* forward: 5′-GCCTGCGAGGAGTTCAAGAAGATC-3′, *Rgs16* reverse: 5′-TGGTCAGTTCTCGGGTCTCGTG-3′). Routine dissociation curves were performed at the end of the qRT-PCR to verify the presence of a single peak corresponding to the required amplicon. The analysis was executed in both technical and biological triplicate. Data from qRT-PCR experiments were analyzed using the “Livak 2^−ΔΔCT^ method” for relative quantification. The threshold cycle number (Ct) values of the calibrator and the samples of interest were normalized using the geometric average of the endogenous housekeeping genes *Ankrd27* and *Rictor* for the injury models (Gambarotta et al., 2014), and *Tbp* for Schwann cell experiments; the Ct average of the uninjured nerves or of the untreated cells were respectively used as calibrators.

### 2.7 Total protein extraction and Western blot analysis

Total proteins were extracted using boiling Laemmli buffer (2.5% sodium dodecyl sulfate, 0.125 M Tris-HCl pH 6.8) and analyzed, as previously described (Gambarotta et al., 2004; El Soury et al., 2023). Protein concentration was evaluated using the Bicinchoninic Acid method on 1:4 diluted proteins to avoid detergent interference. Equal amounts of protein were loaded into each lane of 12% homemade polyacrylamide gels. Primary antibodies used were anti-RGS16 (#PA5-92131, 1:2000; Invitrogen, United States); anti-glyceraldehyde-3-phosphate dehydrogenase (GAPDH; #4300, 1:20.000; Ambion, ThermoFisher Scientific, Europe); secondary antibodies used were horseradish peroxidase-linked anti-rabbit (#7074) and anti-mouse (#7076; both 1:15,000; Cell Signaling Technology, United States).

### 2.8 Statistical analysis

GraphPad Prism 10.2.2 software (Boston, Massachusetts, United States) was used for statistical analysis of qRT-PCR data. After assessing the normal distribution with the Shapiro-Wilk test, the statistical significance was determined by one-way analysis of variance (ANOVA) followed by Dunnett’s multiple comparisons *post hoc* test (Figure 3, panel G), when all samples were compared with the control only or by Bonferroni’s multiple comparisons *post hoc* test (Figure 3, panels A, B, C, E, F when all samples were compared to each other). Data were presented as mean ± SEM; *p ≤ 0.05, **p ≤ 0.01, ***p ≤ 0.001, ****p ≤ 0.0001.

## 3 Results

### 3.1 Computational analysis of single-cell RNA sequencing datasets shows that the expression of *Rgs* family members is regulated after nerve transection

**FIGURE 1:**
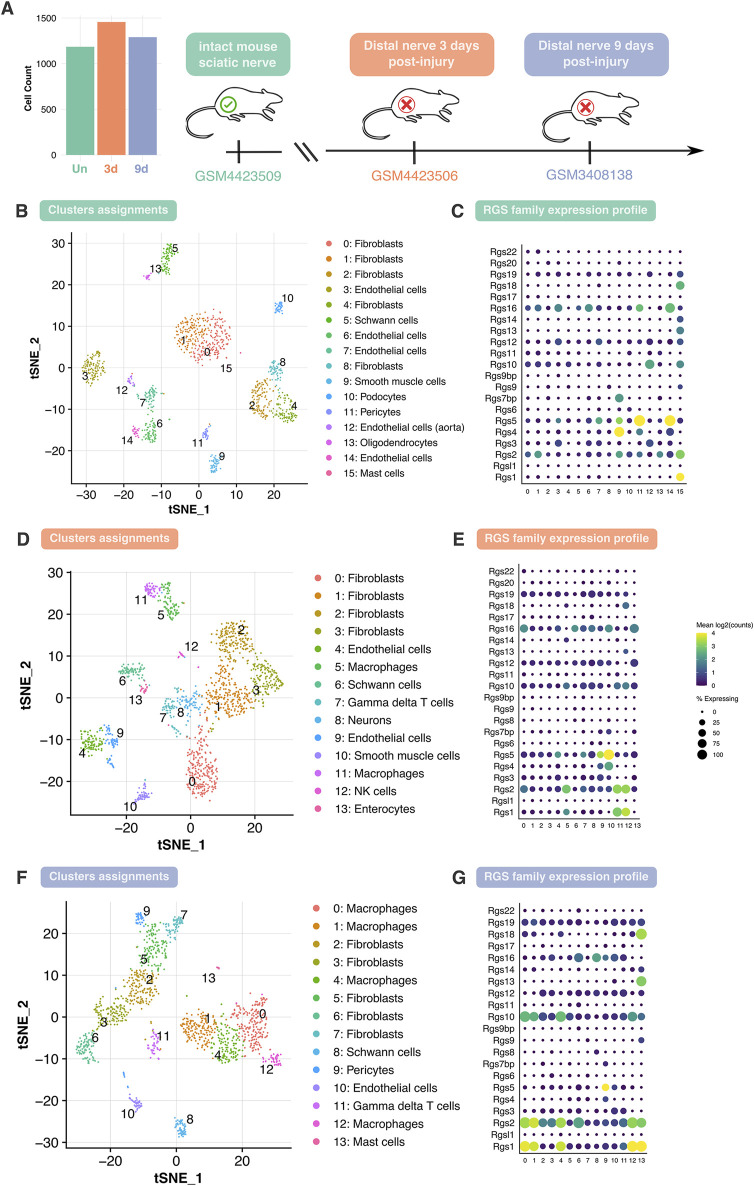
Analysis of cell type heterogeneity and *Rgs* gene family expression in intact and injured mouse peripheral nerves. **(A)** Overview of the analyzed datasets and cell counts. Single-cell RNA sequencing datasets corresponding to three conditions: intact mouse sciatic nerve (Un), distal nerve 3 days post-injury (3d), and distal nerve 9 days post-injury (9d). The bar plot represents the total number of cells analyzed in each dataset, post dataset filtering. **(B, D, F)** t-SNE (t-distributed stochastic neighbor embedding) plot depicting cell clusters identified in Un, 3d and 9d datasets. Cells are grouped into clusters (0–n), with distinct colors representing different cell types, including Schwann cells, as indicated in the legend. **(C, E, G)** Dot plot showing the expression profile of the *Rgs* gene family across different cell clusters in Un, 3d and 9d with dot size indicating the percentage of expressing cells and color representing mean log2 (counts). Abbreviations: t-SNE, t-distributed stochastic neighbor embedding; *Rgs*, regulator of G-protein signaling; Un, uninjured; 3d, 3 days post-injury; 9d, 9 days post-injury.

**FIGURE 2:**
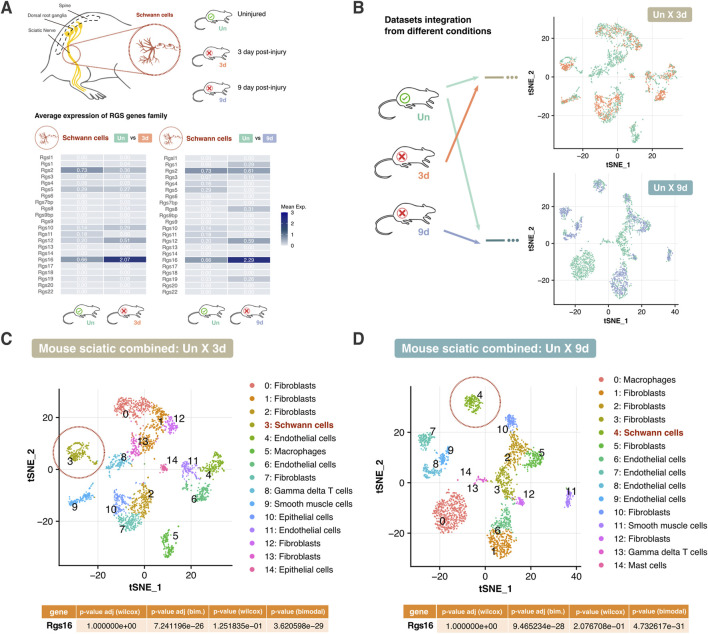
Comparative analysis of *Rgs* gene expression in mouse peripheral nerve cells post-injury. **(A)** Schematic representation of the sciatic nerve and the focus on Schwann cells. The average expression levels of the *Rgs* gene family are shown for uninjured (Un) *versus* 3 days post-transection (3d) and 9 days post-transection (9d) conditions. Heatmaps depict mean expression values for each condition. **(B)** Integration of datasets from different conditions (Un, 3d, 9d) for comparative analysis. t-SNE plots demonstrate the clustering of cell types across conditions. Arrows indicate the integration process between datasets. **(C, D)** t-SNE plot illustrating clustering of cell types in combined intact and 3 days post-injury sciatic nerve, and intact and 9 days post-injury sciatic nerve datasets. Schwann cells are circled in red. The table below presents adjusted p-values from Wilcoxon and Likelihood-ratio (bimodal) tests for *Rgs16*, indicating significant differential expression. Abbreviations: Un, Uninjured; 3d, 3 days post-injury; 9d, 9 days post-injury.

The cellular composition and *Rgs* gene family expression patterns in healthy and injured adult mouse peripheral nerves using scRNA-seq data was examined. To perform the scRNA-seq analysis, three different datasets were chosen: GSM4423509 dataset for uninjured sciatic nerve group (Un) (Toma et al., 2020), GSM4423506 for sciatic nerve 3 days post-transection group (3d) (Toma et al., 2020) and GSM3408138 for sciatic nerve 9 days post-transection group (9d) (Carr et al., 2019). After low-quality cell removal, all datasets maintained comparable high-quality cell counts (Figure 1A). Various cell populations across these datasets were identified, revealing variations in cell-type composition and gene expression over different conditions (Figures 1B–G). Cluster assignment and t-SNE projections were used to stratify the cell types across the datasets, where shifts in cellular populations suggest dynamic responses to nerve injury (Figures 1B, D, F). In addition, the attention was focused on profiling the expression of the *Rgs* gene family. Specifically, the expression of 22 *Rgs* gene family members was examined across the identified cell clusters in all three datasets (Figures 1C, E, G). Notably, many *Rgs* genes were expressed into several nerve cell types (including, among others, nerve fibroblasts, endothelial cells, Schwann cells, and macrophages) in both healthy and injured nerves (Figures 1C, E, G). Focusing attention on Schwann cells—critical players in nerve repair—it could be observed that *Rgs16* expression increased at 3 and 9 days post-injury (Figure 2A).

Since Schwann cell activity is modulated following injury, data from different conditions (Un x 3d and Un x 9d) were integrated, and t-SNE projections were employed to track shifts in gene expression within Schwann cell populations. The distribution of Schwann cells in the 3 days and 9 days post-injury datasets confirmed dynamic transcriptional changes over time. Clustering analysis further revealed clear segregation between uninjured and post-injury Schwann cells, highlighting distinct transcriptional profiles in response to nerve injury (Figure 2B).

Further analysis of Schwann cells uncovered distinct expression patterns between uninjured and injured conditions. Differential gene expression analysis confirmed that *Rgs16* is upregulated in Schwann cells following injury (Figures 2C, D). A complex expression pattern suggested that a subset of Schwann cells upregulated *Rgs16* after nerve injury, while another subcluster showed reduced expression. Such patterns may be overlooked by the Wilcoxon test, which assessed population-wide changes. In contrast, the Likelihood-ratio test modeled the mean and proportion of expression as common across groups. The significantly adjusted p-value indicated a differential expression of *Rgs16* between conditions, suggesting the *Rgs16* potential role as a regulator of Schwann cell activity in nerve degeneration.

### 3.2 *Rgs16* is regulated after traumatic nerve injury and during regeneration

To analyze the regulation of the expression of *Rgs16* after peripheral nerve injury, qRT-PCR analysis was carried out on RNA obtained from the distal portion of injured median nerves belonging to three experimental groups of adult female rats at different time points post-injury (3, 7, 14, and 28 days): crush injury (a mild injury model), end-to-end repair (more severe injury, where a transection is followed by repair), and degenerating nerve (where nerve transection is not followed by repair) and, as control, healthy median nerves.

**FIGURE 3:**
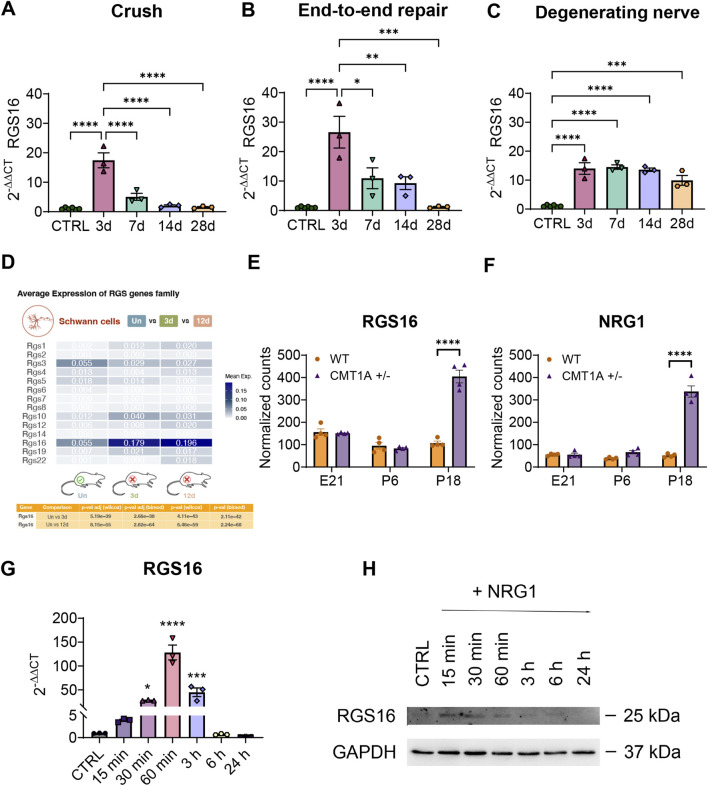
Evaluation of *Rgs16* expression in acute and chronic injury and after NRG1β1 stimulation of Schwann cells. **(A–C)** Quantitative expression analysis of *Rgs16* under regenerating and degenerating conditions following traumatic injury to the median nerve in Wistar rats. The relative quantification of *Rgs16* was evaluated by qRT-PCR at different time points after injury (d = day post-injury). Regenerating conditions: **(A)** crush injury; **(B)** end-to-end repair after transection injury. Degenerating conditions: **(C)** transection injury. The geometric mean of the housekeeping genes *Ankrd27* and *Rictor* was used to normalize data. The healthy nerves were used as calibrator. The values in the graphs are expressed as the mean ± SEM (n = 3-6 each group). **(D)** Comparative analysis of *Rgs* gene expression in rat Schwann cells after chronic constriction injury obtained from single-cell RNA sequencing dataset. The heatmap shows average expression levels of the *Rgs* gene family for uninjured (Un) *versus* 3 days post-injury (3d) and 12 days post-injury (12d) conditions. The table below presents adjusted p-values from Wilcoxon and Likelihood-ratio (bimodal) tests for *Rgs16*, indicating significant differential expression. **(E, F)** Normalized counts of RNA-sequencing data of *Rgs16* and *Nrg1* genes in a model of chronic demyelinating pathology Charcot-Marie-Tooth disease type-1A during development. The values in the graphs are expressed as the mean ± SEM (n = 4 each group). **(G)** Quantitative expression analysis by qRT-PCR of *Rgs16* after 10 nM NRG1β1 stimulation of Schwann cells. *Tbp* was used as a housekeeping gene to normalize data. Untreated cells were used as calibrator. The values in the graph are expressed as the mean ± SEM (n = 3 each group). **(H)** Western blot analysis of RGS16 after 10 nM NRG1β1 stimulation of primary cultures of Schwann cells at different time points.

These results showed that *Rgs16* expression is significantly increased in the injured nerve compared to the healthy control nerve. Specifically, *Rgs16* mRNA expression was upregulated 3 days post-injury, showing a 17-fold increase (P < 0.0001) after crush injury and a 27-fold increase (P < 0.0001) after transection injury followed by end-to-end repair. Following this initial increase, *Rgs16* expression decreased under regenerative conditions, reaching healthy nerve levels (Figures 3A, B). However, in degenerative conditions, expression levels remained elevated from 3 days post-injury, showing a sustained 14-fold increase (P < 0.0001). By the end of the observation period (28 days), expression was still nearly 10-fold (P < 0.0001) compared to controls (Figure 3C). The sustained upregulation of *Rgs16* in the degenerating nerve was confirmed through the analysis of rat scRNA-seq data from chronic constriction injury samples (Lovatt et al., 2022) (Figure 3D).

### 3.3 *Rgs16* is overexpressed in Charcot-Marie-Tooth type-1A pathological condition

The observed upregulation of *Rgs16* following traumatic peripheral nerve injuries suggests a potential involvement for *Rgs16* in chronic demyelinating peripheral neuropathies. To explore this hypothesis, a pathological experimental rat model of Charcot-Marie-Tooth-1A disease (CMT1A), characterized by the overexpression of the myelinating protein PMP22 (Patel et al., 1992; Sereda et al., 1996), was analyzed. RNA sequencing data from the GEO database corresponding to wild-type and CMT1A rats at different developmental stages (E21, P6 and P18) (Fledrich et al., 2018) were examined. Consistent with the hypothesis, *Rgs16* expression was significantly elevated in the transgenic rats, showing a 3.8-fold increase (P < 0.0001) compared to wild-type animals at P18, correlating with the presence of pathological conditions (Figure 3E).

### 3.4 NRG1 treatment stimulates *Rgs16* expression in primary Schwann cell cultures

Neuregulin1 (*Nrg1*), known for its role in Schwann cell dedifferentiation and demyelination, shows marked upregulation in response to both acute and chronic nerve injuries, particularly in its soluble isoform (Carroll et al., 1997; Stassart et al., 2013; Ronchi et al., 2016). In models of CMT1A demyelinating neuropathy (Sereda et al., 1996), soluble *Nrg1* upregulation is evident during nerve development at RNA and protein levels (Fornasari et al., 2018). This upregulation was confirmed (Figure 3F) by the analysis of the previously mentioned RNA sequencing dataset (Fledrich et al., 2018).

To determine whether soluble NRG1 regulates RGS16 expression, rat primary Schwann cell cultures were treated with 10 nM recombinant NRG1β1 and analyzed at various time points (15, 30 and 60 min, 3, 6, and 24 h). At the mRNA level, *Rgs16* expression showed a progressive increase starting 15 min after stimulation, peaking at a 128-fold upregulation (P < 0.0001) at 60 min. Subsequently, *Rgs16* expression gradually declined eventually returning to baseline levels comparable to the control condition (Figure 3G). At the protein level, RGS16 upregulation was detectable 15 min post-treatment, but decreased 60 min after stimulation (Figure 3H).

## 4 Discussion

G protein-coupled receptors (GPCRs) are highly effective pharmacological targets of numerous drugs commonly used in the treatment of a wide range of pathologies such as cardiovascular diseases, psychiatric disorders or allergies (Hauser et al., 2017; Doyen et al., 2020). Notably, GPCRs are abundantly expressed in nervous tissues (Vassilatis et al., 2003; Doyen et al., 2020).

The activity of GPCR is finely regulated by various mechanisms, including modulation by Regulators of G protein signaling (RGS) proteins. The canonical RGS signaling mechanism consists in the activation of the GTPase-activity of the Gα-subunit, which accelerates GTP hydrolysis and effectively deactivates GPCR signaling (Wieland and Chen, 1999; Derrien and Druey, 2001; Larminie et al., 2004; Tian et al., 2022; Yuan and Yang, 2022).

Modulating the activity of specific RGS proteins, either positively or negatively, holds potential for addressing various neuropathologies of the peripheral nervous system (PNS), including nerve injury. Such modulation could enhance regeneration and mitigate long-term pathological consequences. To explore this potential, it is necessary to understand the role of RGS proteins in injured nerves. To this end, a bioinformatic analysis of single-cell RNA-sequencing (scRNA-seq) data was performed to identify genes regulated following peripheral nerves injury. This study revealed that *Rgs* genes are regulated and that *Rgs16*, a member of the R4 RGS family and also known as A28-RGS14 or RGS-R (Bansal et al., 2007; Tian et al., 2022), is the most highly expressed *Rgs* member in Schwann cells of the sciatic nerve at 3 and 9 days after transection. RGS16 is known to be expressed in multiple tissues such as the retina (Chen et al., 1996; Snow et al., 1998), pituitary gland (Chen et al., 1997), liver (Chen et al., 1997), heart (Kardestuncer et al., 1998; Patten et al., 2002), brain (Grafstein-Dunn et al., 2001) and hematopoietic cells (Beadling et al., 1999; Shi et al., 2004; Kveberg et al., 2005; Kim et al., 2006). While the association of RGS16 in immune, inflammatory, tumor, and metabolic disorders is well established (Tian et al., 2022), its potential involvement within the PNS has not yet been investigated.

RGS16 inhibits GPCR activity through the canonical signaling mechanism by acting as GTPase-activating protein. Accordingly, RGS16 overexpression inhibits the activity of CXC chemokine receptor 4 (CXCR4), the receptor of stromal cell-derived factor (SDF1α or CXCL12), in a megakaryocytic cell line (Berthebaud et al., 2005), in follicular dendritic cells (Estes et al., 2004) and in platelets (Karim et al., 2016). Interestingly, CXCR4 is expressed also by motor neurons and by sensory neurons playing a role—respectively—in neuromuscular junction regeneration (Negro et al., 2017) and neuropathy after dorsal root ganglia (DRG) chronic compression (Yu et al., 2017). Another example of the canonical signaling mechanism is the RGS16-mediated regulation of circadian cAMP levels in the suprachiasmatic nucleus. During the day, RGS16 expression is upregulated, and by inhibiting GPCR Gpr176 signaling, it enhances adenylate cyclase activity, thereby promoting cAMP production (Goto et al., 2017).

RGS16 regulates cell signaling not only through canonical mechanisms, but also via non-canonical pathways. For example, RGS16 was demonstrated to interact with the p85α subunit of phosphoinositide 3-kinase (PI3K), inhibiting its interaction with the scaffolding protein Gab1 in the breast cancer MCF-7 cell line (Liang et al., 2009); RGS16 inhibits Gα-mediated signaling not by enhancing GTPase activity but by directly binding to Gα13 and this interaction blocks Gα13 association with its substrate, p115Rho-GEF, thereby modulating Rho activation (Johnson et al., 2003); RGS16 overexpression suppresses SDF1α-induced activation of mitogen-activated protein kinase (MAPK) and protein kinase B (AKT) in the megakaryocytic MO7e cell line (Berthebaud et al., 2005).

To confirm the bioinformatic findings from mouse scRNA-seq analysis showing *Rgs16* involvement in peripheral nerve injury, three experimental rat models of nerve injury and repair were examined at multiple time points, providing a broader perspective beyond the nerve transection model, including not only nerve degeneration, but also nerve regeneration. The obtained data showed a significant upregulation of *Rgs16* mRNA expression 3 days post-injury, with a sharp decline observed by day 7 in regenerative conditions. Conversely, in degenerative conditions, *Rgs16* expression remained consistently elevated throughout the experimental timeline (3, 7, 14 and 28 days after nerve transection in rat), coherently with the results obtained in the scRNA-seq (3 and 9 days after nerve transection in mouse). Analysis of a scRNA-seq dataset of degenerating rat nerves (3 and 12 days after chronic constriction injury) confirmed these findings. This evidence suggested that RGS16 may act as an immediate response factor following nerve injury, with its expression sustained in the absence of nerve regeneration.

After assessing *Rgs16* expression in degenerating conditions in different models of traumatic nerve injury, its expression was evaluated in a rat model of chronic degeneration, Charcot-Marie-Tooth type-1A (CMT1A), an inherited peripheral demyelinating neuropathy affecting motor and sensory nerves. CMT1A is characterized by distal muscle atrophy, weakness, decreased nerve conduction velocity, and hypertrophic neuropathy. CMT1A is caused by the duplication of the peripheral myelin protein 22 (*Pmp22*) gene, which is expressed in Schwann cells and primarily localized in the compact myelin (Lupski et al., 1991; Raeymaekers et al., 1991; Patel et al., 1992). The bioinformatic analysis of wild-type and CMT1A rat datasets (Fledrich et al., 2018) showed that *Rgs16* and Neuregulin1 (*Nrg1*) are overexpressed during development in peripheral nerves of transgenic rats.

In fact, nerves affected by CMT1A exhibit overexpression of the soluble isoform of NRG1 (Fornasari et al., 2018), a factor released by Schwann cells after injury and involved in demyelination and Schwann cell dedifferentiation (Carroll et al., 1997; Zanazzi et al., 2001; Stassart et al., 2013; Ronchi et al., 2016). Different NRG1 isoforms exist (Mei and Xiong, 2008): transmembrane isoforms are expressed by axons and are involved in myelination; soluble isoforms are produced by Schwann cells and nerve fibroblasts (Fornasari et al., 2020) as precursor transmembrane proteins. The soluble NRG1 active fragment is released in the extracellular environment after proteolytic cleavage mediated by a disintegrin and metalloprotease 17 (ADAM17), also called tumor necrosis factor-α-converting enzyme (TACE) (Mei and Xiong, 2008).

The concomitant upregulation of *Nrg1* and *Rgs16* following both traumatic injury and chronic degeneration suggests a potential regulatory relationship, where NRG1 may influence RGS16 expression and, conversely, RGS16 might modulate NRG1-mediated signaling.

To investigate the potential crosstalk between RGS16 and NRG1, primary Schwann cells were stimulated with recombinant NRG1β1. Following Schwann cell stimulation, RGS16 expression increased both at RNA and protein level, thus confirming a possible link between RGS16 and NRG1 during nerve degeneration after injury and in demyelinating chronic conditions.

RGS16 expression is upregulated following nerve injury, potentially playing a pivotal role in early Schwann cell dedifferentiation, activation, and migration. As a key modulator of GPCR signaling, RGS16 may fine-tune cellular responses essential for promoting efficient nerve regeneration.

GPCRs can transactivate tyrosine kinase receptors; in particular, it has been shown that type 1A Angiotensin II receptor transactivates the Epidermal growth factor receptor (EGFR/ErbB1) and this transactivation is inhibited by metalloproteinase inhibitors (Thomas et al., 2002) or ADAM17/TACE small-interfering RNA (Wang et al., 2009). These results suggest that GPCR-ErbB transactivation might be mediated by ADAM17/TACE activation, followed by the proteolytic cleavage of ligand precursors (Strachan et al., 2010; Palanisamy et al., 2021). Since soluble NRG1 release is also regulated by ADAM17/TACE (Mei and Xiong, 2008; Palanisamy et al., 2021), we could speculate that GPCRs might regulate NRG1 release and, therefore, transactivate NRG1 receptors, namely, ErbB3 and ErbB4. Conversely, an inhibitory crosstalk from ErbB4 receptors to GPCRs has been identified, wherein NRG1 induces a reduction in 5-hydroxytryptamine 2A GPCR activity in vascular smooth muscle cells, embryonic fibroblasts, and cortical pyramidal neurons. This mechanism suggests a feedback system that might attenuate GPCR signaling via the effector protein p90 ribosomal S6 kinase 2 (RSK2) (Strachan et al., 2010).

**FIGURE 4:**
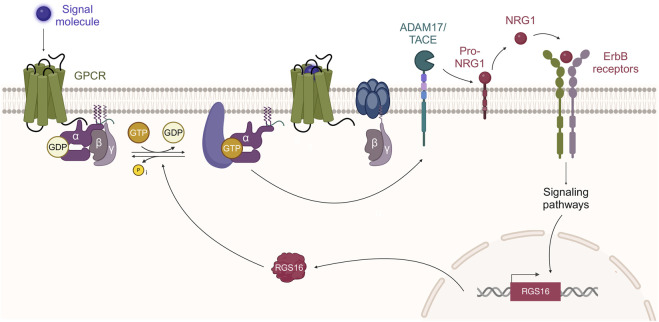
GPCR-ErbB transactivation and RGS16 mediated negative feedback loop. It has been proposed that GPCR stimulation, followed by Gα activation, can activate ADAM17/TACE activity (Palanisamy et al., 2021). ADAM17/TACE, by proteolytic cleavage, promotes the release of soluble NRG1 that activates ErbB receptors. NRG1 stimulation of ErbB receptors (ErbB3-ErbB2 in Schwann cells) promotes RGS16 transcription and translation. RGS16, which is a GTPase activating protein, promotes GTP dephosphorylation, followed by Gα protein inactivation. Created by MG-B in BioRender https://BioRender.com/a81c288.

We hypothesize that in Schwann cell primary cultures, and potentially in injured nerves, NRG1 might attenuate GPCR signaling by upregulating RGS16, which subsequently would inactivate Gα proteins. In turn, RGS16, as a negative modulator of Gα signaling downstream of GPCR, might participate in a negative feedback loop, ultimately switching off ADAM17/TACE-mediated proteolytic NRG1 release (Figure 4).

RGS16 might not only be involved in nerve degeneration, but also in neuropathic pain. Our data show that RGS16 is overexpressed in CMT1A disease, characterized by this symptomatology. Neuropathic pain research has been mainly focused on neuronal mechanisms; nevertheless, emerging evidence indicates that glial cells are key contributors to both the onset and resolution of pain. While most studies focused on glial cells in the central nervous system, the involvement of Schwann cells in neuropathic pain is less understood. However, recent research highlights the important role of Schwann cells to detect nerve injury and to develop and maintain neuropathic pain (Wei et al., 2019). GPCRs are expressed in Schwann cells and play a key role in regulating pain. Among them, lysophosphatidic acid receptor expression is necessary to develop neuropathic pain after peripheral nerve injury (Inoue et al., 2004). In addition, γ-aminobutyric acid B (GABA-B) receptors are expressed in Schwann cells (Magnaghi et al., 2004), and Schwann cell conditional knock-out mice are hyperalgesic and allodynic (Faroni et al., 2014); indeed, GABA-B stimulation promotes nerve regeneration and ameliorates neuropathic pain (Magnaghi et al., 2014).

Interestingly, some RGS members are involved in neuropathic pain; for example, RGS4 absence correlates with recovery from mechanical and cold allodynia (Avrampou et al., 2019). RGS proteins have shown therapeutic potential in neurodegenerative conditions like Parkinson’s disease (Lee and Bou Dagher, 2016; O’Brien et al., 2019; Ahlers-Dannen et al., 2020), multiple sclerosis (Lee and Bou Dagher, 2016), and pathological itch sensation (Pandey et al., 2017), therefore their targeted modulation could offer a promising strategy for treating neuropathic pain. In this context and considering the existence of pro- and anti-nociceptive GPCRs, RGS might have a negative or positive impact on endogenous pain modulators as well as on drugs used in the treatment of pain syndromes (Doyen et al., 2020). By fine-tuning the expression of specific RGS proteins, it might be possible to reduce neuropathic pain by altering GPCR signaling, which could lead to more precise and effective treatments with fewer side effects compared to current pain management therapies. In fact, the inhibition of RGS proteins with small molecule inhibitors is known to provide analgesia (Senese et al., 2020).

High-throughput screening techniques have facilitated the identification of several RGS inhibitors over the past few years (O’Brien et al., 2019). However, there are currently no approved drugs specifically designed to target RGS16. Various therapeutic strategies, including small interfering RNA, small-molecule inhibitors, and CRISPR-based approaches, hold potential for targeting or modulating RGS16 activity. While our findings offer initial insights, the precise role of RGS16 in nerve degeneration and regeneration remains unclear, making it challenging to hypothesize its direct therapeutic application for nerve injury treatment. Further investigation is required to define its function and therapeutic relevance.

In conclusion, the observed temporal expression of *Rgs16* suggests a transient regulatory role in response to NRG1 signaling, potentially influencing key cellular mechanisms involved in peripheral nerve repair or related pathological processes. A major limitation of this study is the lack of *in vivo* functional validation confirming the link between NRG1 and RGS16. Future research is essential to fully unravel the role of RGS16 in peripheral nerve degeneration and regeneration, as well as its potential contribution to neuropathic pain modulation. A deeper understanding of these mechanisms could pave the way for novel therapeutic approaches aimed at enhancing nerve repair and alleviating chronic pain conditions.

## Data Availability

The raw data supporting the conclusions of this article will be made available by the authors, without undue reservation.
