# Supplementary material for: Dynamic regulation of Rgs16 and its correlation with Neuregulin1 expression in acute and chronic nerve injury
Source: Front Cell Dev Biol. 2025 Mar 27;13:1540453. doi: 10.3389/fcell.2025.1540453 (PMC11983618; doi:10.3389/fcell.2025.1540453)
Supplement: Supplementary file 1 [file DataSheet1.pdf]

Supplementary Material

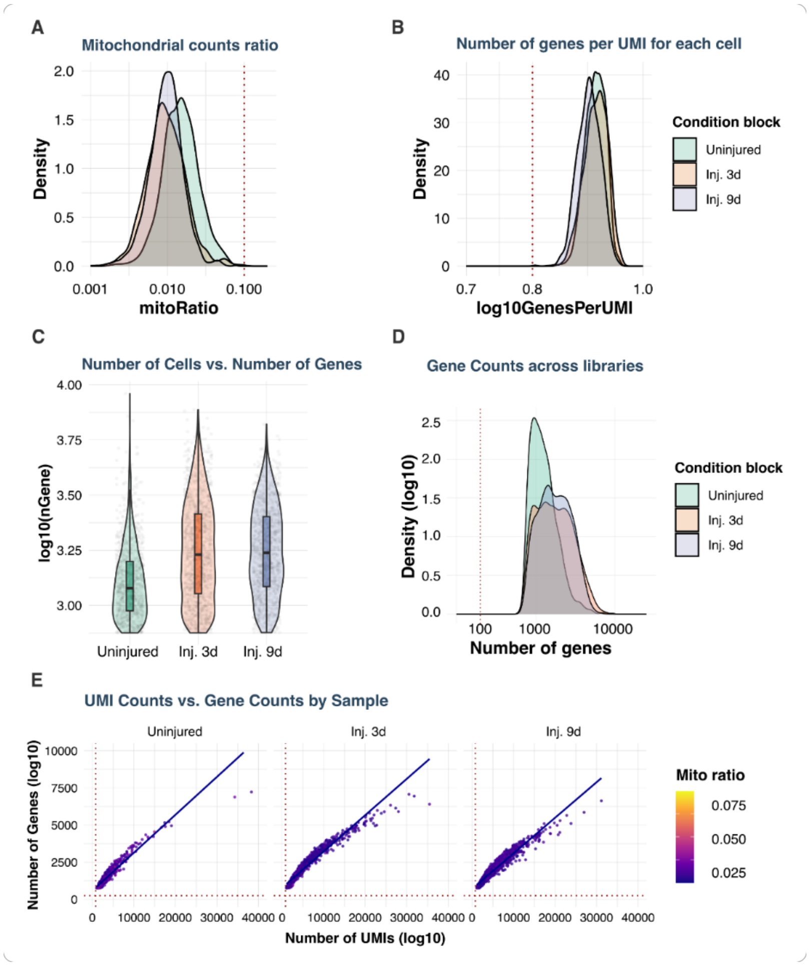

Supplementary Figure 1. Single-cell RNA-sequencing data quality control across intact and injured mouse peripheral nerve datasets. (A) Density plot showing the distribution of the ratio of mitochondrial gene counts to total counts (mitoRatio) across conditions. The dashed red line represents the typical threshold for filtering out cells with high mitochondrial content, indicating potential cell stress, damage or death. (B) Density plot showing the distribution of the number of genes per unique molecular identifier (UMI) across datasets. The dashed red line marks the lower limit for acceptable gene complexity per cell, ensuring cells with low gene counts are filtered. (C) Violin plot comparing the distribution of the number of detected genes across cells from each condition. Differences are highlighted in gene detection levels between uninjured and injured states, with the median and interquartile range indicated by boxplots within each violin. (D) Density plot illustrating the distribution of total gene counts across different cells and conditions. (E) Scatter plots show the relationship between UMI counts and detected gene counts for cells from each dataset. The blue regression line represents the expected positive correlation between UMI and gene counts, with points color-coded by mitochondrial ratio. The red dashed lines indicate quality control thresholds (th) for minimum UMI (th = 800) and gene counts (th = 250).
